# Supplementary material for: LWD–TCP complex activates the morning gene CCA1 in Arabidopsis
Source: Nat Commun. 2016 Oct 13;7:13181. doi: 10.1038/ncomms13181 (PMC5065627; doi:10.1038/ncomms13181)
Supplement: Supplementary Information — Supplementary Figures 1-14, Supplementary Tables 1-4, Supplementary Note 1 and Supplementary References. [file ncomms13181-s1.pdf]

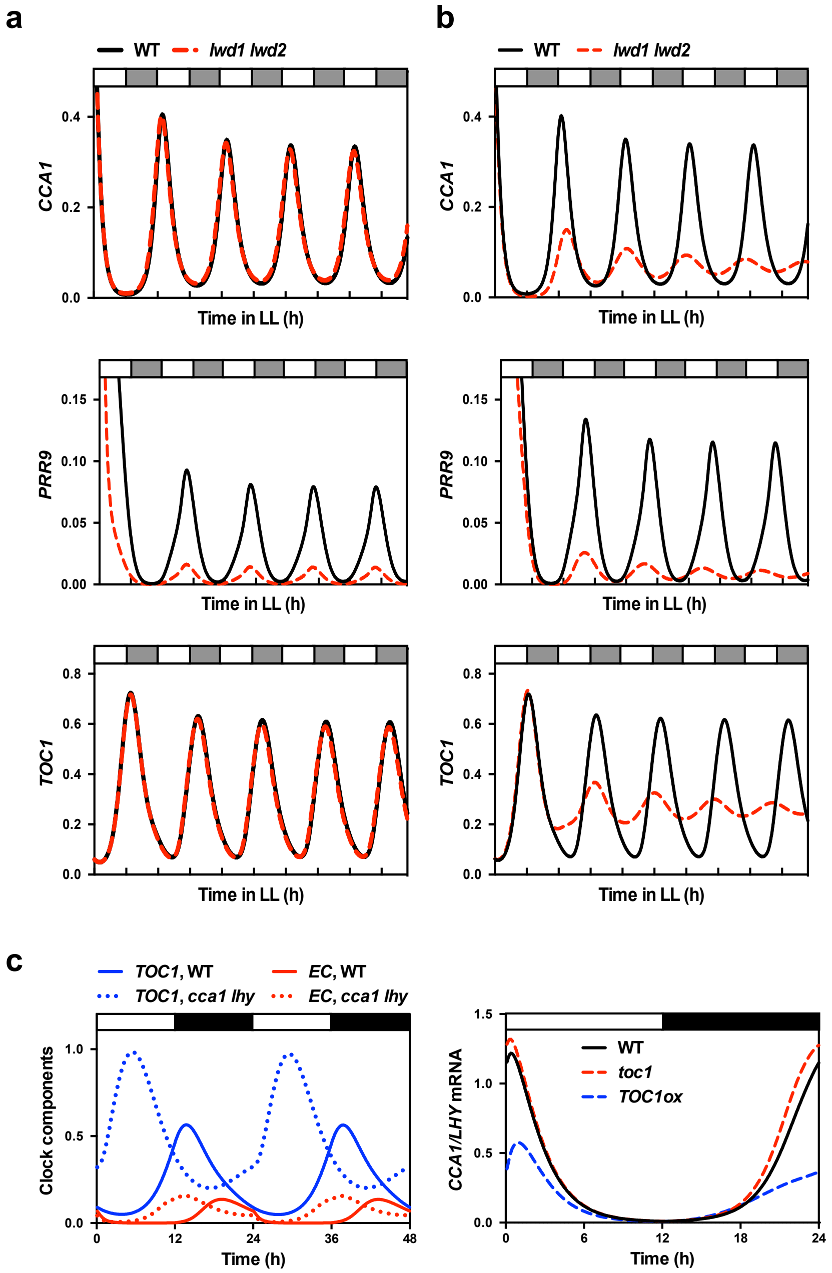

Supplementary Fig. 1. The integration of LIGHT-REGULATED WD1 (LWD1) sustained the robust performance of the Pokhilko model

The Pokhilko model was revised to incorporate LWD1 as an activator for *PRR9* only (a) or both *PRR9* and *CCA1* (b). The mRNA expression profiles of *CCA1*, *PRR9* and *TOC1* in both the wild type (WT) and *lwd1 lwd2* mutant under constant light are shown. (c) Genetic perturbation tests were simulated in *cca1 lhy*, *toc1* and *TOC1ox* for the expression of *TOC1, EVENING COMPLEX* (*EC*) and *CCA1/LHY* with the revised Pokhilko model implementing LWD1 in this study.

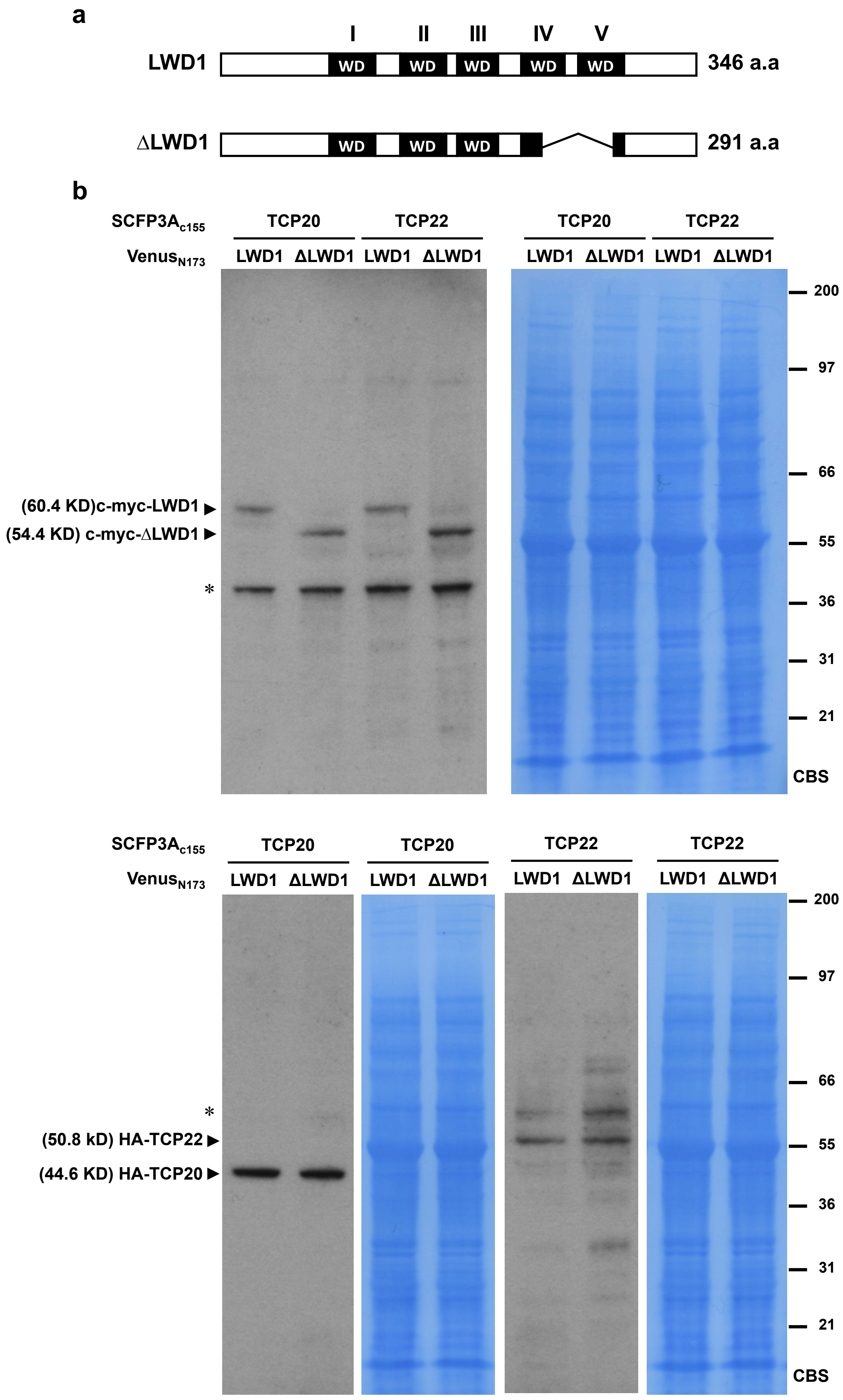

Supplementary Fig. 2. Protein expression of LWD1/ΔLWD1 and TCP20/TCP22 by bimolecular fluorescence complementation (BiFC) assay

(a) Construct diagrams of full-length LWD1 and truncated LWD1 (ΔLWD1) used for transient expression in *Arabidopsis* seedlings. The WD repeat motifs I to V are in black boxes. (b) Protein lysates were prepared from seedlings co-cultured with *Agrobacteria* harboring four BiFC combinations. Immunoblotting involved use of anti-LWD1 and anti-HA antisera to detect c-myc-LWD1 and HA-TCP20/22, respectively. Asterisks indicate the non-specific bands. Coomassie blue-stained (CBS) membranes are shown for equal protein loading.

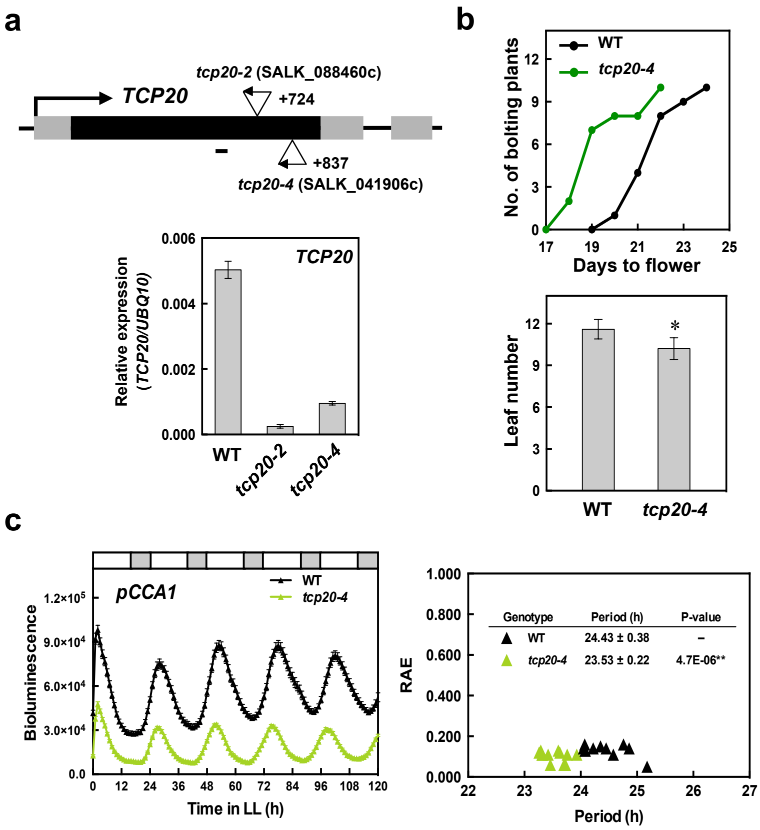

Supplementary Fig. 3. Molecular characterization of *tcp20* mutants

(a) Schematic representation of *tcp20* T-DNA insertion lines. The insertion sites (relative to the translation start +1) were sequence-validated. qRT-PCR of *TCP20* mRNA levels in the WT and *tcp20-2* and *tcp20-4* mutants normalized to *UBQ10* expression (*TCP20/UBQ10*). Black horizontal line indicates the region amplified on qRT-PCR. (b) Early flowering of *tcp20-4* mutant under long-day conditions. Data are mean ± SD (n ≥ 10). Asterisk indicates that mutant plants flowered significantly earlier than WT plants (Student's t test; *P<0.01). Ten to 12 plants for each genotype were planted for scoring for each biological replicate. Similar results were observed in three independent experiments. (c) *pCCA1::LUC2* level in *tcp20-4* mutant. Reduced period length in *tcp20-4* mutant (n ≥ 11 seedlings per genotype). Similar results were observed in three independent experiments and one representative result was shown. Data are mean ± SE. Period length and relative amplitude error (RAE) were calculated by FFT-NLLS analysis according to data from LL48 to LL120. Asterisks indicate that period lengths were significantly shortened in *tcp20-4* (Student's t test; **P<0.001).

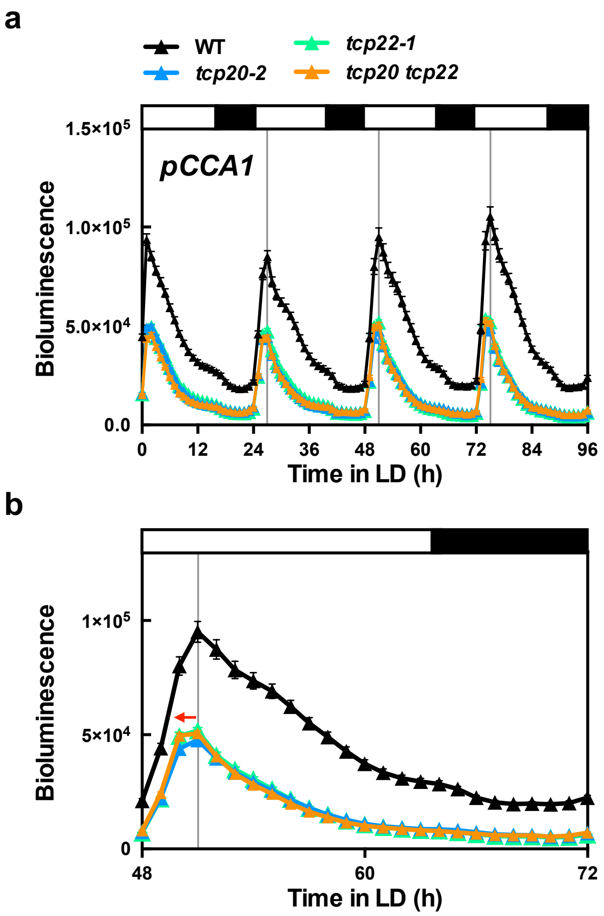

Supplementary Fig. 4. Reduced and phase-advanced expression of *pCCA1::LUC2* in *tcp* mutants under entrainment conditions

(a) Reduced expression amplitudes of *pCCA1::LUC2* in *tcp20-2*, *tcp22-1* and *tcp20 tcp22* mutants under 16h-light/8h-dark conditions. (b) Advanced expression (arrow) of *pCCA1::LUC2* in the *tcp* mutants in a representative 24-h cycle (48-72 h). Vertical line marks the expression peak of *pCCA1::LUC2* in WT plants. Data are mean ± SE (n ≥ 15 seedlings for each genotype).

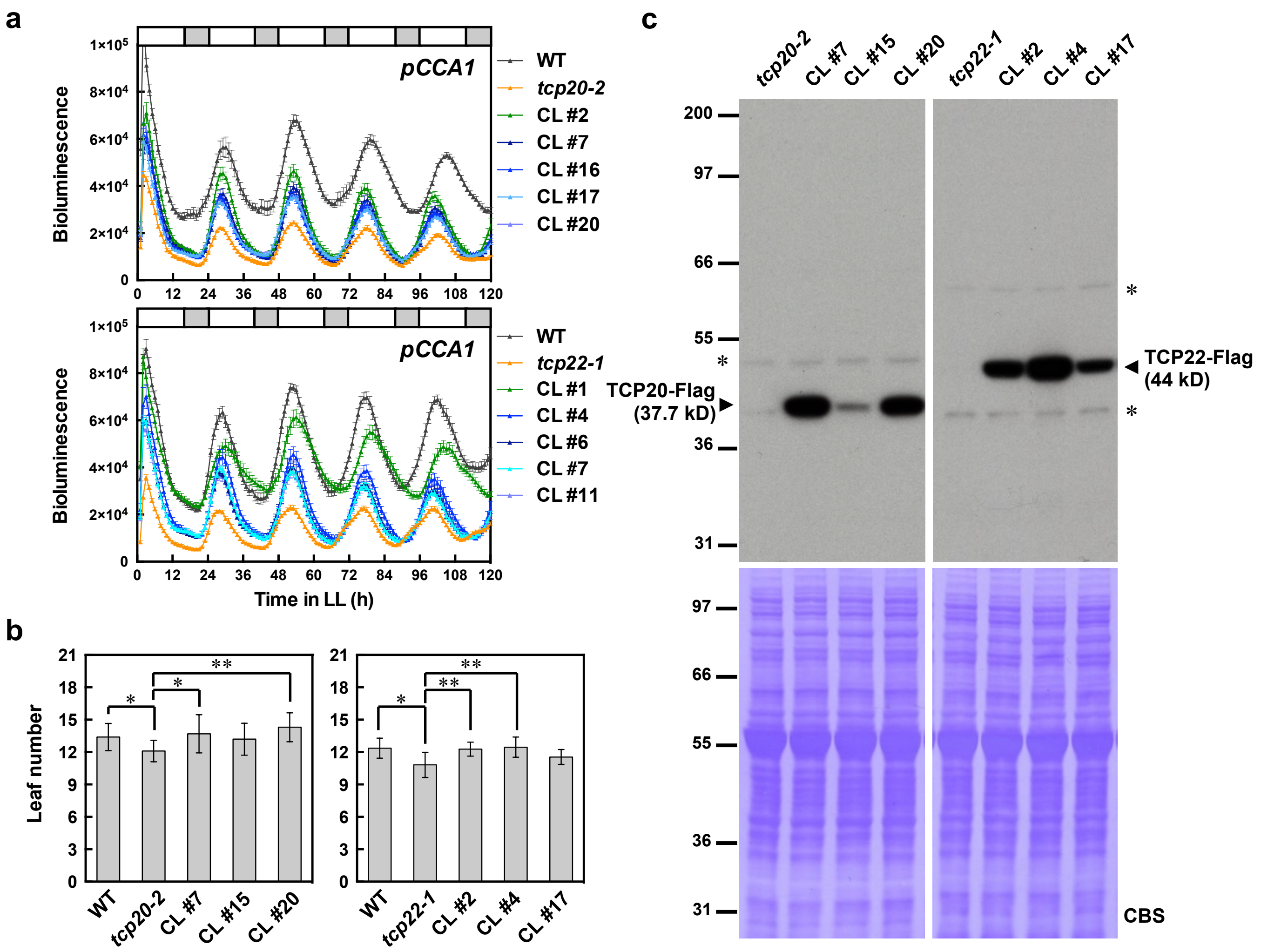

Supplementary Fig. 5. Functional complementation of *tcp20-2* and *tcp22-1* mutants

(a) The *CCA1* promoter activities in *tcp20-2* and *tcp22-1* mutants were partially complemented by expressing *pTCP20::TCP20-Flag* and *pTCP22::TCP22-Flag*, respectively. Seven-d-old seedlings of *tcp20-2 pTCP20::TCP20-Flag pCCA1::LUC2*, *tcp22-1 pTCP22::TCP22-Flag pCCA1::LUC2* and corresponding reporter line in the WT grown under 16-h light/8-h dark (75 μmol m^{-2} s^{-1}) were transferred to continuous light (30-35 μmol m^{-2} s^{-1}) at ZT0 and imaged every hour for 5 days. T2 plants from five independent complementation lines (CLs) for each complementation assay were analyzed. Data are mean ± SE (n = 6-8). (b) *pTCP20::TCP20-Flag* and *pTCP22::TCP22-Flag* complemented the early flowering phenotype in *tcp20-2* and *tcp22-1* mutants, respectively, under long-day conditions. Data are mean ± SD (n ≥ 10). Asterisks indicate significantly different flowering time between *tcp* mutants and the WT or between *tcp* mutants and CLs (Student's t test; *P<0.05, **P<0.01). Ten to 12 plants for each genotype were planted for scoring for each biological replicate. Similar results were observed in three independent experiments. (c) Seven-d-old seedlings grown under long-day conditions were collected at ZT10 for immunoblot analyses with anti-Flag antiserum. Asterisks indicate the non-specific bands. Portions of the Coomassie blue-stained (CBS) blot are shown for protein loading control.

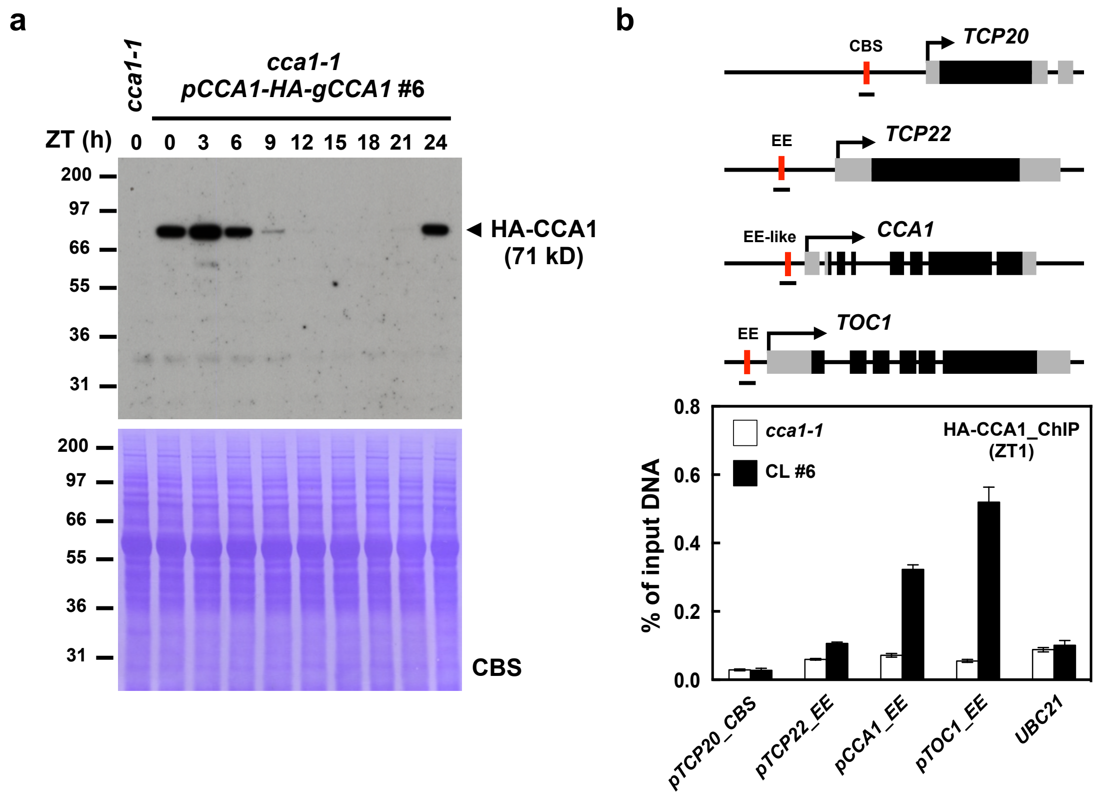

Supplementary Fig. 6. CCA1 does not bind with CCA1-binding site (CBS) or evening element (EE) in *TCP20* or *TCP22* promoters

(a) Anti-HA antibody was used to examine the expression of HA-CCA1 driven by the native promoter in the complementation line *cca1-1 pCCA1::HA-gCCA1* #6 (CL #6) under long-day conditions. Three biological replicates showed similar patterns of HA-CCA1 accumulation. (b) ChIP-qPCR assay of the binding of HA-CCA1 to the indicated promoters with anti-HA antibody in *cca1-1* mutant and in CL#6. Red vertical bars are the EE, EE-like and CBS in the promoter regions of the indicated genes. Horizontal bars are amplicons for ChIP-qPCR analyses. Data are mean ± SD from one representative experiment (n = 3). Similar results were observed in three independent experiments.

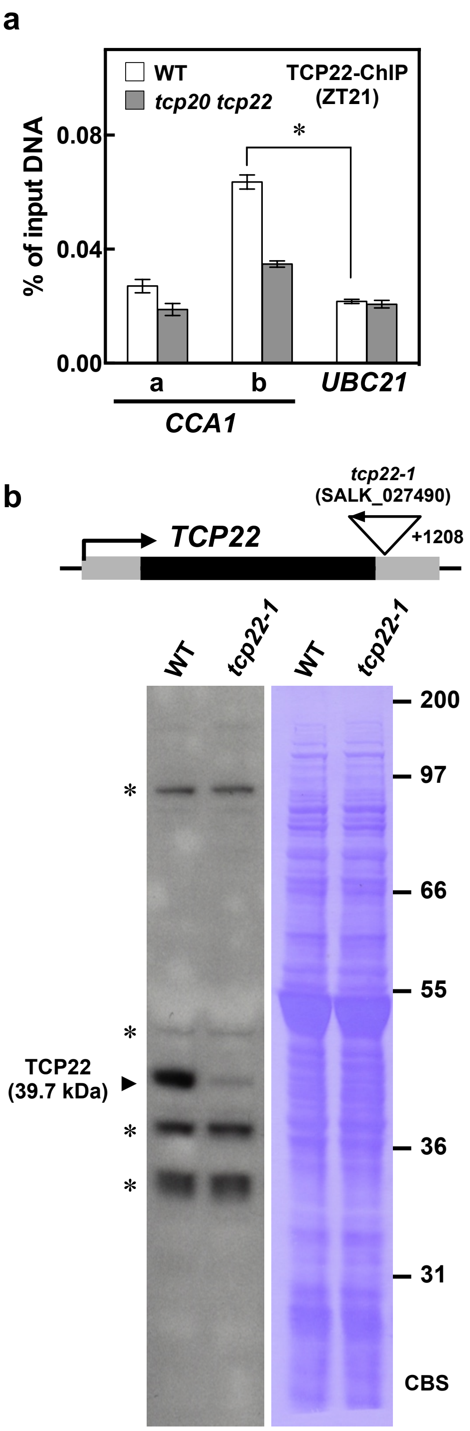

Supplementary Fig. 7. TCP22 associates with TCP-binding site (TBS)-containing region of *CCA1* promoter *in vivo*.

(a) TCP22 associates with TBS-containing region of *CCA1* promoter *in vivo*. ChIP assays involved use of anti-TCP22 antisera. Data are mean ± SD (n = 3). Asterisk indicates that TCP22 preferentially binds to the amplicon 'b' (Student's t test; *P<0.0005). Similar results were observed in two independent experiments. (b) Schematic representation of T-DNA insertion in *tcp22-1* used in this study. The insertion site at +1208 (relative to the translation start +1) was sequence-validated. Gray and black boxes indicate the untranslated and coding regions. Rosette leaves of 40-d-old WT and *tcp22-1* grown under 16-h light/8-h dark conditions were collected at ZT4 for immunoblot analyses. Polyclonal antisera against TCP22 raised in rabbit recognized a protein of approximately 40 kDa which is greatly decreased in the *tcp22-1* mutant. Asterisks indicate the non-specific bands. A portion of the Coomassie blue-stained (CBS) blot is shown for protein loading control.

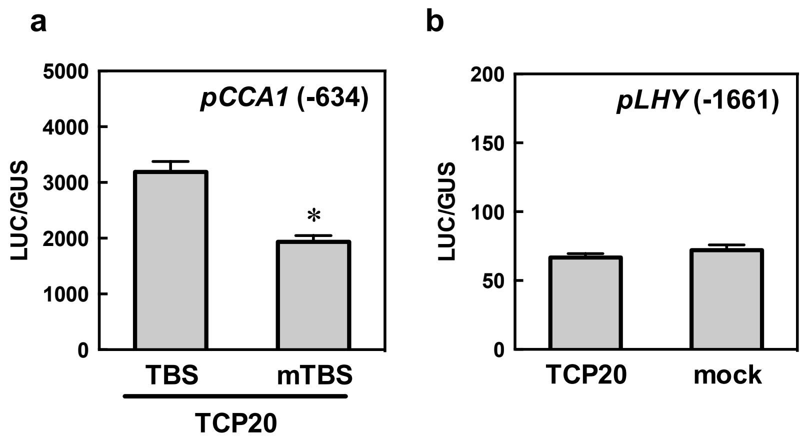

Supplementary Fig. 8. TCP20 activates *CCA1* promoter but not *LHY*

(a) Bioluminescence analyses of the reporter *LUC2* driven by WT TBS (TBS) or mutated TBS (mTBS) version of *pCCA1* (-634) involved TCP20 as the effector in *Arabidopsis* protoplasts. Asterisk indicates that *pCCA1* activity is significantly reduced when TBS is mutated (Student *t* test; *P <0.001). (b) TCP20 did not activate the expression of *pLHY* (-1661*)*. LUC2 activity was normalized to GUS activity from the transfection control *35S::GUS*. Mock represents an empty effector vector co-transfected with the reporter construct. Data are mean ± SD (n = 3). Similar results were observed in three independent experiments.

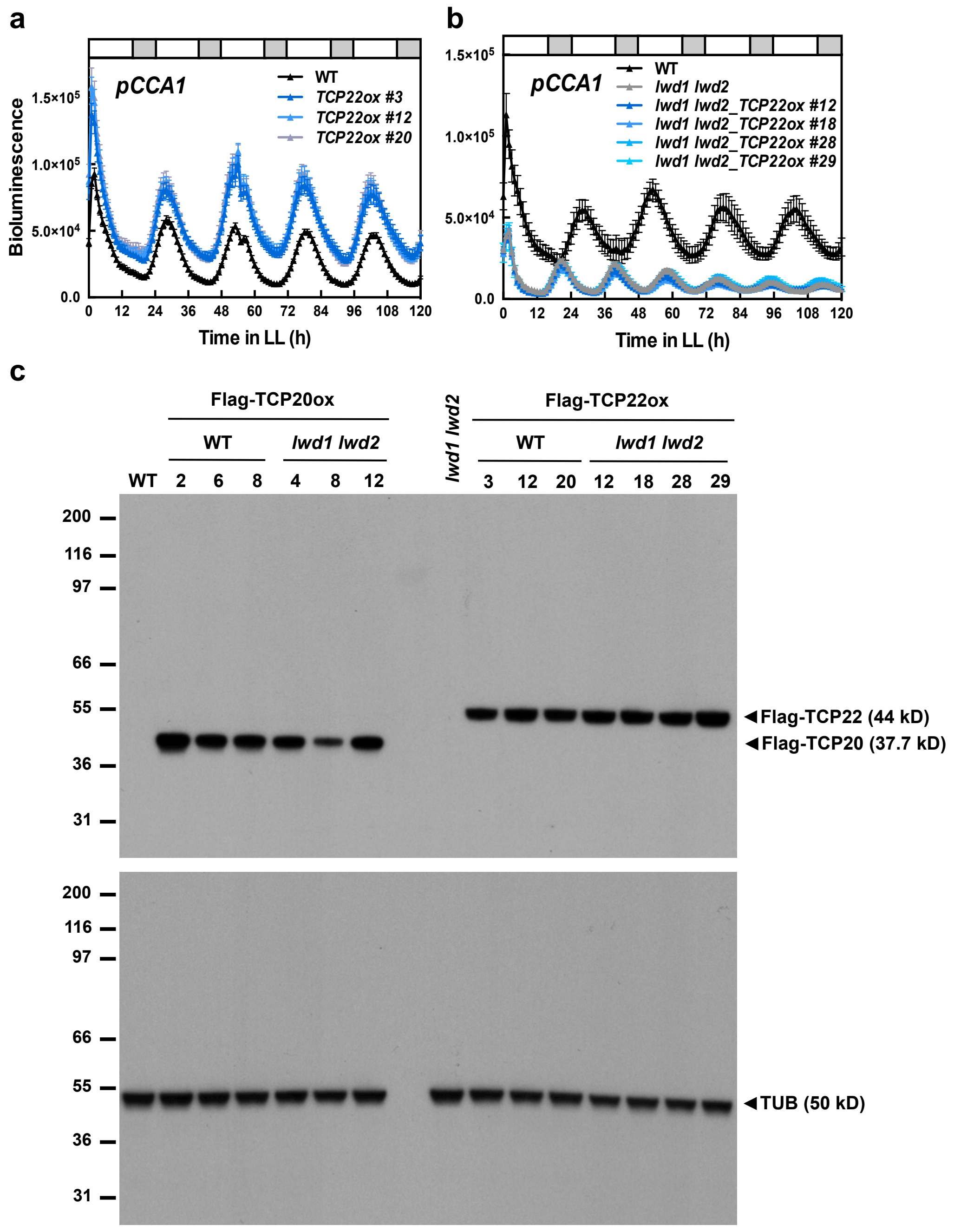

Supplementary Fig. 9. TCP22 activates *CCA1* in an LWD-dependent manner

(a) *pCCA1::LUC2* expression is increased in TCP22-overexpressing lines. (b) TCP22 overexpression in an *lwd1 lwd2* background fails to increase the expression of the *CCA1* promoter. Bioluminescence of *pCCA1::LUC2* expression in the WT and *lwd1 lwd2*. Data are mean ± SE (n = 6 to 8). Similar results were observed in three independent experiments. (c) TCP20/TCP22 protein accumulation in *TCP20ox/ TCP22ox* lines in the WT or with an *lwd1 lwd2* background. Immunoblot analyses involved use of anti-Flag and anti-tubulin antisera to detect Flag-TCP20/TCP22 and internal control tubulin, respectively.

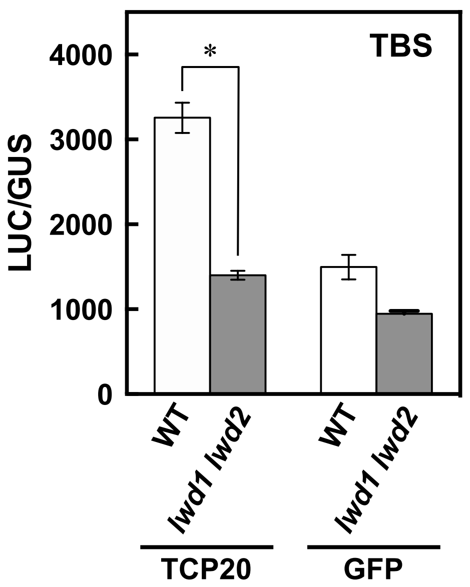

Supplementary Fig. 10. Functional dependency of TCP20 on LWD1

(a) TCP20 possessing activator activity for the TBS of *CCA1* promoter in transient assays with protoplasts isolated from WT plants but not *lwd1 lwd2* mutants. Data are mean ± SD (n = 3). Asterisk indicates that the TCP20 activator activity toward TBS is significantly increased in WT (Student's t test; *P<0.005). Similar results were observed in two independent experiments.

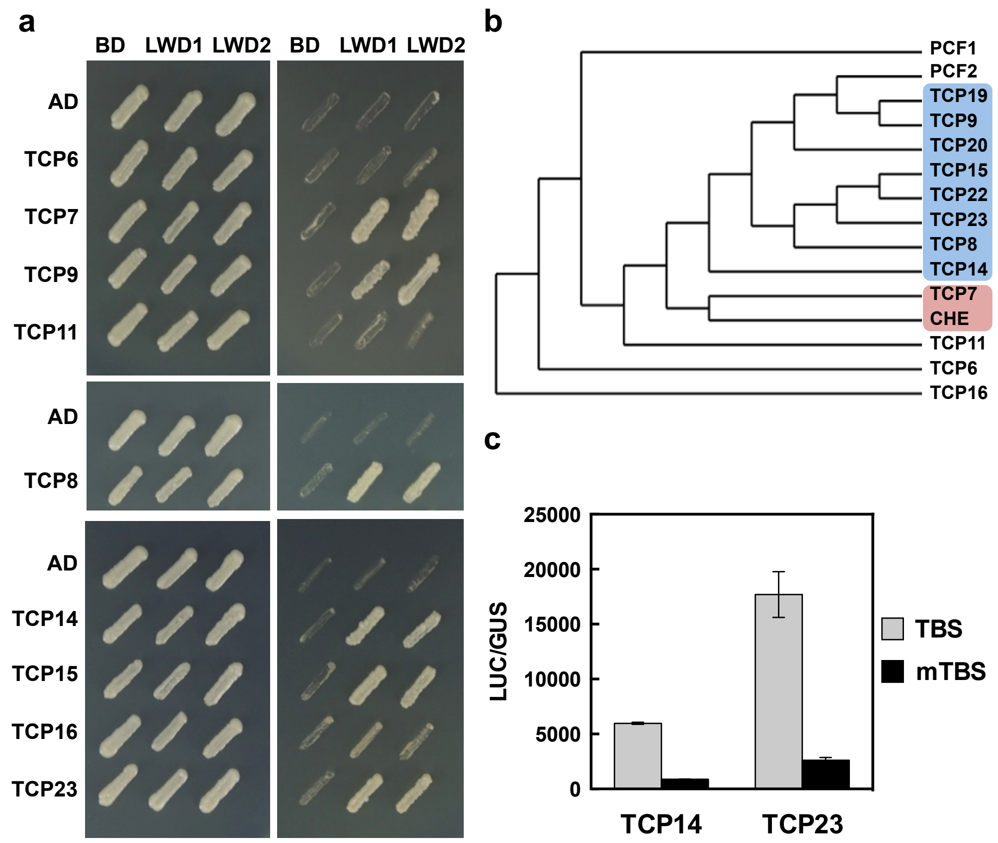

Supplementary Fig. 11. LWDs interact with additional class I TCP members that are transcriptional activators

(a) LWD1/LWD2 interact with other class I TCP members on yeast two-hybrid assays. Bait and prey constructs selected on the SD-WL and LWD–TCP interaction was assessed on SD-WLH with 3-AT of 0.5 mM for TCP6/TCP7/TCP9/TCP11, 3 mM for TCP8, and 2 mM for TCP14/TCP15/TCP16/TCP23. (b) Phylogenetic analysis of the relationship among class I TCP members. The clades for LWD-interacting TCP members are in blue or red. CHE is a repressor of *CCA1*. PCF1 and PCF2 are TCP transcription factors from rice. (c) Transient assay in *Arabidopsis* protoplasts of TCP14 and TCP23 possessing activator activity toward the promoter containing TBS but not mTBS. Data are mean ± SD (n = 3). Similar results were observed in three independent experiments.

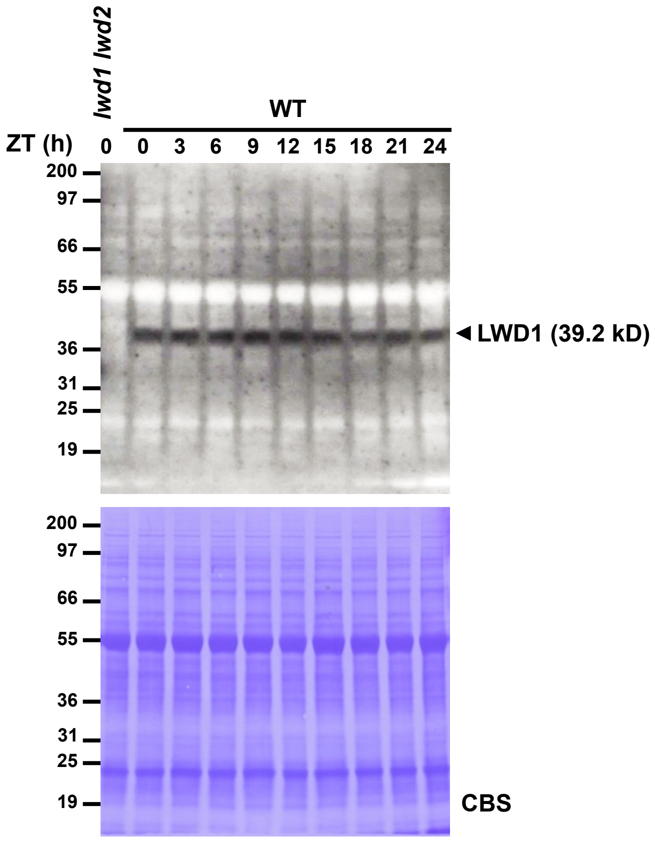

Supplementary Fig. 12. Accumulation of LWD1 protein in a 24-h cycle

LWD1 protein level was determined in the WT by using anti-LWD1 antibody. An amount of 30 µg total protein prepared from 14-d-old seedlings was loaded for each sample collected at the indicated ZT under the long-day conditions (16-h light/8-h dark). A portion of the Coomassie blue-stained (CBS) blot is shown for protein loading control. Data are representative of three biological replicates with similar patterns of LWD1 accumulation.

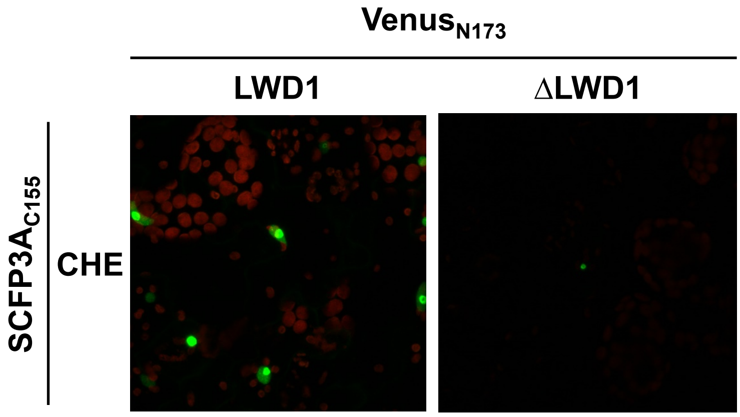

Supplementary Fig. 13. LWD1 interacts with the *CCA1* repressor CHE

The BiFC assays were performed using Venus$_{N173}$-fused full-length (LWD1) or truncated (ΔLWD1) with SCFP3A$_{C155}$-fused CHE. The fluorescence signal detected in nuclei with the LWD1 and CHE interaction was reduced with the combination of ΔLWD1 and CHE.

Supplementary Fig. 14. Results of biological replicates in this study

Biological replicates of Fig. 1b

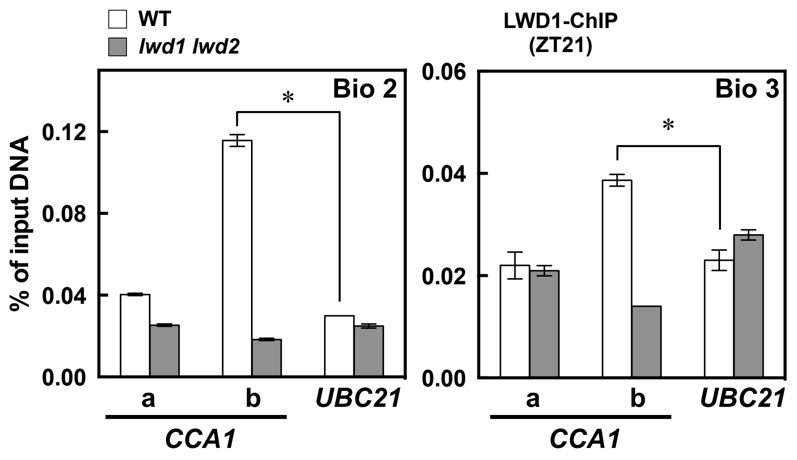

Biological replicate of Fig. 3a

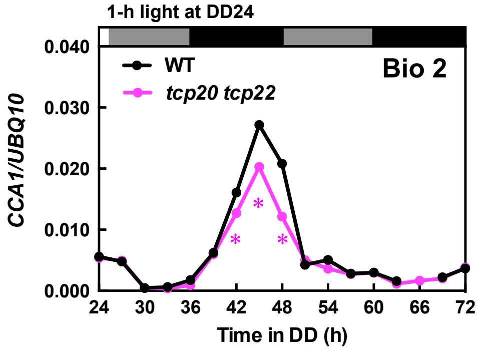

Biological replicates of Fig. 3e

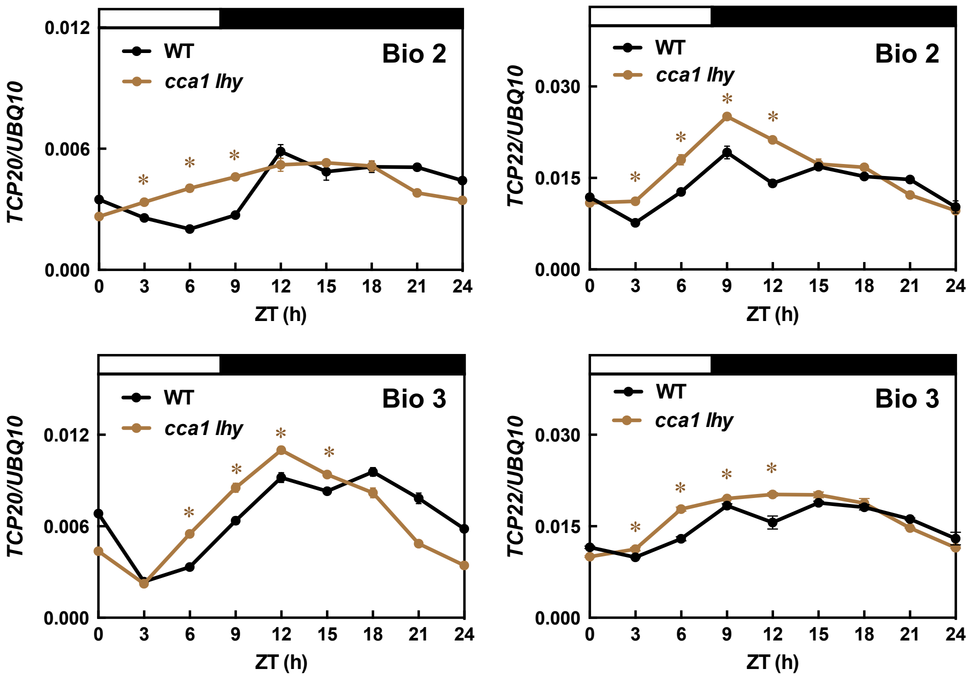

Biological replicate of Fig. 4a

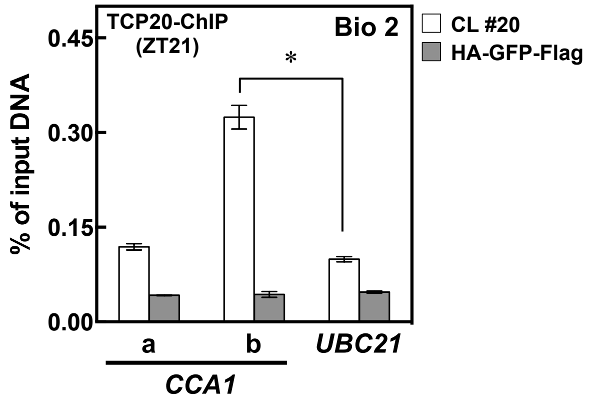

Biological replicates of Fig. 5c

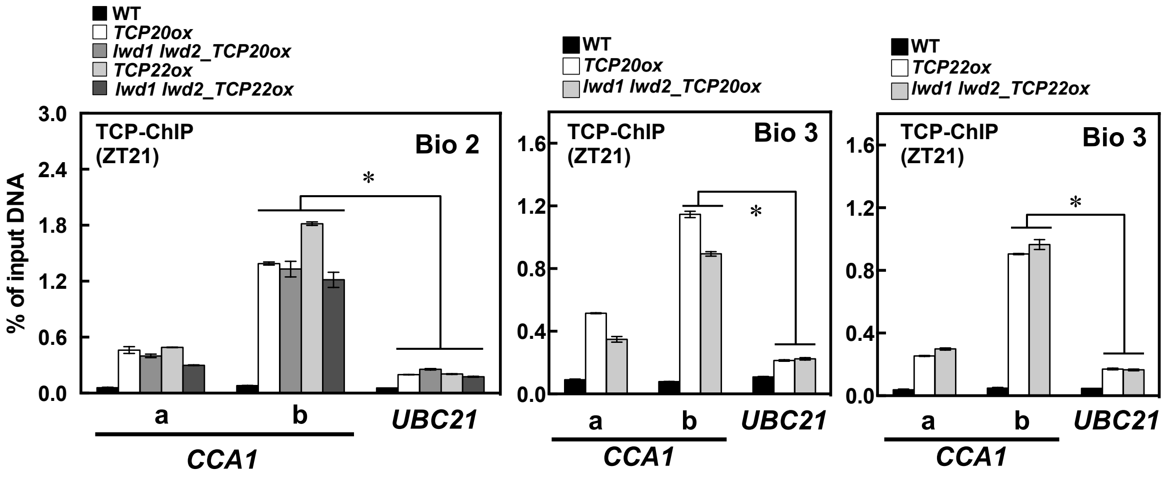

Biological replicates of Fig. 5d

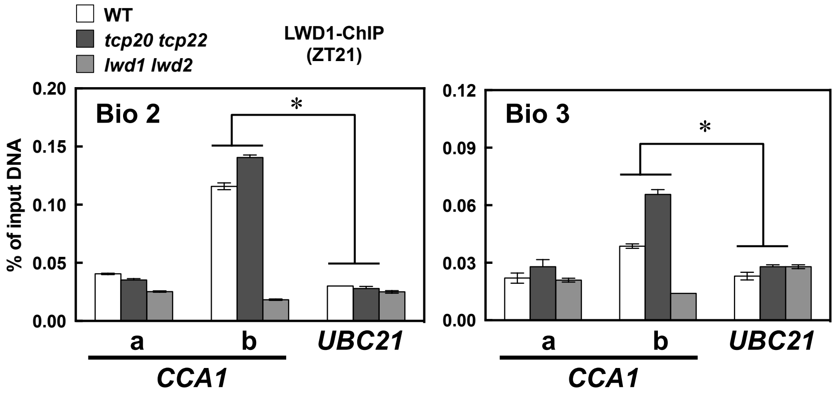

Supplementary Table 1

LWD1 interacts with TCP family members in yeast two-hybrid assays

TCP family	LWD1 interaction prey		Positive transformant count			
	AGI No.	Gene name	3-AT (mM)			Total
			1.0	0.5	0.1	
Class I	At1g72010	*TCP22*	92	89	58	239
	At3g27010	*TCP20*	1	1	80	82
	At5g51910	*TCP19*	0	0	2	2
	At5g08330	*TCP21/CHE*	0	0	1	1
Class II	At1g53230	*TCP3*	0	0	2	2

Supplementary Table 2

Primers and oligos used in this study

Name	Sequence (5' to 3')
**Promoter::LUC2 constructs**	
pTCP20-PstI-Fw	AAACTGCAGAAACGATTCCAATTAGCCTC
pTCP20-SalI-Rev	AGGCGTCGACCGAAGCTTGCTTGTGTGG
pTCP22-PstI-Fw	ATACTGCAGATGGGTTTAGAAGGAGTCAT
pTCP22-SalI-Rev	TGGCGTCGACCTTCAAATCCGTAAAAAGATATG
pCCA1 (-984)-PstI-Fw	CTCTGCAGGTCTCTGGTCTTTTTTAG
pCCA1 (-634)-PstI-Fw	TGTCTGCAGGTCCACTGATGTTTCTAGTGT
pCCA1 (-1)-NcoI-Rev	TCAACCATGGCACTAAGCTCCTCTACACAA
pLHY(-1661)-PstI-Fw	CTTCTGCAGGATTCGGGTAGTTCAGTTCTT
pLHY(-1)-SalI-Rev	GATGGTCGACAACAGGACCGGTGCAGCTATT
**Yeast two-hybrid**	
SalI-T7 promoter-Fw	ACCTGTCGACCTTTAATACGACTCACTAT
PstI-NotI-Rev	GTTAGCGGCCGCACTACGATTCATCTGCAGC
LWD1-XmaI-Fw	TACGCCCGGGTATGGGAACGAGCAGCGAT
LWD1-NotI-Rev	ATAAGCGGCCGCTCAAACCCTGAGAATTTGCA
LWD2-XmaI-Fw	TCAGCCCGGGAATGGTTACGAGCAGCGAT
LWD2-NotI-Rev	CTTTGCGGCCGCTCAGACCCGGAGAATCTG
TCP6-NdeI-Fw	CAGCATATGGTCATGGAGCCCAAGAAG
TCP6-NotI-Rev	TATGCGGCCGCTTATGAACCATTTTCCTCT
TCP7-NdeI-Fw	CTTCATATGTCTATTAACAACAACAACA
TCP7-NotI-Rev	ATAGCGGCCGCTTAACGTGGATCTTCCTCTC
TCP8-NdeI-Fw	CAGCATATGGATCTCTCCGACATCCGA
TCP8-NotI-Rev	TATGCGGCCGCTCACTCAGAGCTATTTGAG
TCP9-NdeI-Fw	TAACATATGGCGACAATTCAGAAGCTTG
TCP9-NotI-Rev	TTAGCGGCCGCTCAGTGGTTCGATGACCGTG
TCP11-NcoI-Fw	TAGCCATGGAGATGATTTTTCAGAATGTGTGCA
TCP11-NotI-Rev	TATGCGGCCGCCTAATGGTGACGGCGTCTA
TCP14-NdeI-Fw	CTCCATATGCAAAAGCCAACATCAAG

TCP14-NotI-Rev	TATGCGGCCGCCTAATCTTGCTGATCCTC
TCP15-NdeI-Fw	CTTCATATGGATCCGGATCCGGATCATA
TCP15-NotI-Rev	TTTGCGGCCGCCTAGGAATGATGACTGGTGC
TCP16-NdeI-Fw	CAGCATATGGATTCGAAAAATGGAATTA
TCP16-NotI-Rev	TTTGCGGCCGCTCAAACTGTGGTTGTGGCTG
TCP20-NdeI-Fw	CGCTCATATGGATCCCAAGAACCTAAA
TCP20-XmaI-Rev	TCGCCCGGGTTAACGACCTGAGCCTTG
TCP22-NdeI-Fw	AGTCCATATGAATCAGAATTCCTCTGT
TCP22-XmaI-Rev	TCCCCCGGGTCACTTTTTGTCATCACC
TCP23-NdeI-Fw	CTTCATATGGAGTCCCACAACAACAACC
TCP23-NotI-Rev	TTTGCGGCCGCTCAAGGAGAACCATCTAT

BiFC

CHE-BamHI-Fw	CGGGATCCATGGCCGACAACGACGGA
CHE-stop-SmaI-Rev	TCCCCCGGGTCAACGTGGTTCGTGGTCGT
LWD1-SpeI-Fw	GGACTAGTATGGGAACGAGCAGCGAT
LWD1-stop-SmaI-Rev	TCGCCCGGGTCAAACCCTGAGAATTTG
LWD1-BamHI-Fw	TACGGATCCTCAAGCTTTGATTTGGGAT
TCP20-SpeI-Fw	GGACTAGTATGGATCCCAAGAACCTA
TCP20-stop-SmaI-Rev	TCGCCCGGGTTAACGACCTGAGCCTTG
TCP22-SpeI-Fw	GGACTAGTATGAATCAGAATTCCTCT
TCP22-stop-SmaI-Rev	TCCCCCGGGTCACTTTTTGTCATCACC

EMSA

TCP-binding-Fw	GAGATTAACGATCTTAAGTAGGTCCCACTA
TCP-binding-Rev	TTCGTTATAATATCTTGATCTAGTGGGACC
TCP-binding-Fw (mut)	GAGATTAACGATCTTAAGTATTGAAACATA
TCP-binding-Rev (mut)	TTCGTTATAATATCTTGATCTATGTTTCAA

ChIP-qPCR

pCCA1-a-Fw	TGTCAAAGTGTTGTAAATTCCTCAAGA
pCCA1-a-Rev	GCATGAAGGGTAGAAGACTAAATGG

pCCA1-b-Fw	TCGACAAACTGGTGGGAGAG
pCCA1-b-Rev	TCCGGGACTACCTGAAAGGTT
pTCP20-CBS-Fw	AGGGATTAATTTTCTACACATTGT
pTCP20-CBS-Rev	GGTAACAATCCAATAACAGTTGAT
pTCP22-EE-Fw	TGAACAACCAACAAATCTCACAC
pTCP22-EE-Rev	AGACTACGTGATGTGTACTGTTT
pTOC1-EE-Fw	TTTGTTGATTTTGATATGGAGATGC
pTOC1-EE-Rv	GGTTGTGTTGGATAGTTTGGTTGAG
UBC21-Fw	TTCAAATGGACCGCTCTTATCA
UBC21-Rev	AAACACCGCCTTCGTAAGGA
**qRT-PCR**	
UBQ10-ABI-Fw	AGAAGTTCAATGTTTCGTTTCATGTAA
UBQ10-ABI-Rev	GAACGGAAACATAGTAGAACACTTATTCA
CCA1-ABI-Fw	CTGTGTCTGACGAGGGTCGAA
CCA1-ABI-Rev	ATATGTAAAACTTTGCGGCAATACCT
TCP20-ABI-Fw	TGGCGGTGAAGGAGTTTCTAGG
TCP20-ABI-Rev	TTGGCACACCAGAACCAAACCC
TCP22-ABI-Fw	ATGCTTCCGATGAGCGGTT
TCP22-ABI-Rev	CGTCCTGTCCCAACTGGATAAT

Supplementary Table 3

Search ranges for parameters

Parameters	Range	Units	Search scale
γ_{cca}, γ_{prr}, γ_{toc} [1]	0.01–10	1/h	Logarithm
α_{prr1}, α_{cca1} [2]	0–1	Dimensionless	Linear
κ's (in all Hill functions)	0.001–1	Dimensionless[1]	Logarithm

[1] β's are set to the same value as the corresponding γ's for a dimensionless unit for the concentration of each gene and for a reduction in number of parameters.

[2] Without loss of generality, we set $\alpha_{prr2} = 1 - \alpha_{prr1}$ (both models) and $\alpha_{cca2} = 1 - \alpha_{cca1}$ (Model II).

Supplementary Table 4

The obtained parameters used in equations (12) and (14)

Model	Parameters	Value	Dimension
I (LWD1 activates *PRR9*)	n_{basal}	0.3	1/h
	n_{lwd}	0.25	1/h
	κ_{lwd}	0.112	Dimensionless
II (LWD1 activates *PRR9* and *CCA1*)	*PRR9* n_{basal}	0.3	1/h
	PRR9 n_{lwd}	0.25	1/h
	PRR9 κ_{lwd}	0.112	Dimensionless
	CCA1 n_{basal}	0.45	1/h
	CCA1 n_{lwd}	4.5	1/h
	CCA1 κ_{lwd}	0.088	Dimensionless

Supplementary Note 1

Mathematical circuit constructions

The operation of the *Arabidopsis* circadian clock was simulated by the inclusion of LWD1 while simplifying the core clock components. *CCA1* and *LHY* genes have a similar expression profile and similar functions[1], so only *CCA1* was considered in our model. *PRR9* and *PRR7* function similarly but consecutively in repressing *CCA1* and *LHY* expression during the day[2,3]. Here, we considered only *PRR9* in the model settings. Since LWD1 showed no significant oscillation (Supplementary Fig. 12), it is treated as a constant input in this simulation. We have validated that this simplification does not alter the insight we obtained when our findings were integrated into a more complex model[4] and yielded results comparable to the previous report (Supplementary Fig. 1).

Model I: LWD1 regulates the circadian clock via activating only *PRR9*

Model I is described as a set of three ordinary differential equations (ODEs) [equations (1) to (3)].

$$\frac{d[cca]}{dt} = \left(\alpha_{cca1}\beta_{cca}\right)Hill_{rep\_prrs}Hill_{rep\_toc} + \left(\alpha_{cca2}\beta_{cca}\right)L\cdot cP - \left(\mu_{cca1}\gamma_{cca} + \mu_{cca2}\gamma_{cca}L\right)[cca] \quad (1)$$

$$\frac{d[prr]}{dt} = \left(\alpha_{prr1}\beta_{prr} + \alpha_{prr2}\beta_{prr}Hill_{act\_lwd}\right)\cdot Hill_{act\_cca} + \left(\alpha_{prr3}\beta_{prr}\right)L\cdot cP - \left(\mu_{prr1}\gamma_{prr} + \mu_{prr2}\gamma_{prr}D\right)[prr] \quad (2)$$

$$\frac{d[toc]}{dt} = \beta_{toc}Hill_{rep\_cca} - \gamma_{toc}[toc] \quad (3)$$

Here, [*cca*], [*prr*], and [*toc*] denote a dimensionless concentration of *CCA1*, *PRR9*, and *TOC1*, respectively. In equations (1) to (3), β_x denotes the total production rate for gene *X*, and γ_x is for the total degradation rates. α's and μ's are dimensionless fractions of total rates from different regulation sources. For example, μ_{cca1} and μ_{cca2} are the fraction for the basal degradation and the additional degradation in the light condition for *CCA1*. *L* represents the light function (*L* = 1 when light is present and *L* = 0 otherwise) and *D* represents darkness (*D* = 1 − *L*). *CCA1* and *PRR9* rapidly accumulate in response to light[5, 6]. Here, we followed previous work[4, 7, 8] and modeled this acute light response by using a light-sensitive activator protein *cP*. The expression

of this hypothetical protein accumulates in the dark and degrades in the light. $Hill_{act}$ and $Hill_{rep}$ are the Hill input functions for an activator and a repressor, respectively:

$$Hill_{rep} = \frac{\kappa^n}{\kappa^n + \left[repressor\right]^n} \quad (4)$$

$$Hill_{act} = \frac{\left[activator\right]^n}{\kappa^n + \left[activator\right]^n} \quad (5)$$

where κ represents the threshold of activator or repressor and n the Hill coefficient that governs the steepness of the input function. The larger the n, the more step-like it is in the input functions.

In the present work, only the free-running condition under constant light is simulated. With $L = 1$, $D = 0$, and cP approaches zero after a long time of constant light[4, 7, 8], equations (1) to (3) can be simplified as equations (6) to (8), in which we have omitted unnecessary fractions such as μ_{cca1} and μ_{cca2}.

$$\frac{d[cca]}{dt} = \beta_{cca} Hill_{rep\_prrs} Hill_{rep\_toc} - \gamma_{cca}[cca] \quad (6)$$

$$\frac{d[prr]}{dt} = \left(\alpha_{prr1}\beta_{prr} + \alpha_{prr2}\beta_{prr} Hill_{act\_lwd}\right) \cdot Hill_{act\_cca} - \gamma_{prr}[prr] \quad (7)$$

$$\frac{d[toc]}{dt} = \beta_{toc} Hill_{rep\_cca} - \gamma_{toc}[toc] \quad (8)$$

We further reduced the parameter space by fixing the value of maximum steady-state concentration of each component to unity and turning it into a dimensionless quantity; in this way, the maximum production rates (β's) and total degradation rates (γ's) are set to be equal. The time t in this model is in the unit of hours, achieved by re-scaling all the time-related parameters so that the period of oscillation in wild type becomes 24 h.

All of the 9 independent parameters were obtained by random search, propagated, and screened for regular oscillation, except for the Hill coefficients (n) which are fixed at 3; the search was performed at a logarithmic scale across three orders of magnitude, for γ's and κ's, and a linear scale for α's. Each parameter was varied with their minimum or maximum values as shown in Supplementary Table 3. The criteria we used were as follows:

(1) The trajectory must oscillate regularly, which was defined by examining the

period and amplitude change in each cycle. We calculated the relative difference of period and amplitude change for each cycle, defined as |(*x*1-*x2*)|/min(*x*1, *x*2), where *x*1 and *x*2 are the period or amplitude calculated from two consecutive cycles. An acceptable regular oscillation has less than 5% relative change for more than 10 cycles.

(2) In the *lwd1 lwd2* mutant, the oscillation must have reduced amplitude (>50%) and shorter period (<21 h), as reported previously[9].

(3) To avoid nonphysical sensitivities to small changes in the simulation, the parameter set must generate similar results from two different ODE solvers (ODE15s and ODE23s).

Model II: LWD1 regulates the circadian clock by activating both *PRR9* and *CCA1*

In this model, equation (1) is modified as equation (9) for light/dark cycling or further as equation (10) for constant light, whereas the other two equations remain the same. There are two additional parameters (α_{cca1} and the corresponding κ value in the new Hill function $Hill_{act\_lwd}$ for *CCA1*) in this model. The parameters were screened similarly to Model I.

$$\frac{d[cca]}{dt} = \left(\alpha_{cca1}\beta_{cca} + \alpha_{cca2}\beta_{cca}Hill_{act\_lwd}\right)Hill_{rep\_prrs}Hill_{rep\_toc} + \left(\alpha_{cca3}\beta_{cca}\right)L\cdot cP_{fun} - \left(\mu_{cca1}\gamma_{cca} + \mu_{cca2}\gamma_{cca}L\right)[cca] \tag{9}$$

$$\frac{d[cca]}{dt} = \left(\alpha_{cca1}\beta_{cca} + \alpha_{cca2}\beta_{cca}Hill_{act\_lwd}\right)Hill_{rep\_prrs}Hill_{rep\_toc} - \gamma_{cca}[cca] \tag{10}$$

Genetic perturbation test

The genetic perturbation test involved changing their total production rate (β) while keeping the same total degradation rate (γ). Here, we systematically scanned for the fraction of production rate for each gene (from 0 to 1, with 0.01 increment), where "0" represents the null mutant condition and "1" the wild-type condition for each parameter set. Depending on the parameter sets selected, a different period estimation is produced (longer/shorter than 24 h). In addition, each parameter set may have a different sensitivity to genetic perturbation; thus some parameter sets can still oscillate under strong genetic perturbation and others cannot.

For the genetic perturbation tests shown in Fig. 1a, we applied the strongest genetic perturbation level while keeping at least two-third of the parameter sets obtained previously that can oscillate regularly under this perturbation. For Model I, the genetic perturbation levels are 0.92 for *toc1* (with 18 parameter sets), 0.83 for *prr9* (with 19 parameter sets), and 0.47 for *cca1* (with 18 parameter sets). For Model II, the genetic perturbation levels are 0.68 for *toc1* (with 695 parameter sets), 0.77 for *prr9* (with 872 parameter sets), and 0.51 for *cca1* (with 769 parameter sets).

Comparison with the Pokhilko model

The latest published model by Pokhilko et al., in 2012[4] (hereafter the Pokhilko model), was modified to test the activator role of LWD1 by keeping most parameters the same as they were originally described. First, we treated LWD1 as an activator of *PRR9* for testing whether this activation was already sufficient to generate previous observations. We incorporated LWD1 into the Pokhilko model by changing *PRR9* from equation (11) to equation (12). Here, the parameters n4 and n7 were removed/replaced because the LWD1 effect was likely indirectly included. Since we want to explicitly explain the LWD1 activation, we added three additional parameters (n_{basal}, n_{lwd}, and K_{lwd}) for describing LWD1 activation to *PRR9*.

$$\frac{dC_{p9}^{m}}{dt} = L.q_3.C_p + \frac{g_8}{g_8 + C_{EC}}(n_4 + n_7.\frac{C_L^e}{g_9^e + C_L^e}) - m_{12}C_{p9}^{m} \qquad (11)$$

$$\frac{dC_{p9}^{m}}{dt} = L.q_3.C_p + (n_{basal} + n_{lwd}\frac{lwd^2}{lwd^2 + K_{lwd}^2})\frac{g_8}{g_8 + C_{EC}}.\frac{C_L^e}{g_9^e + C_L^e} - m_{12}C_{p9}^{m} \qquad (12)$$

The Hill coefficient was fixed as 2, following that used in the Pokhilko model. Next, a random search was performed to obtain the three additional parameters. The search involved the same range as used previously (Supplementary Table 3), with some minor manual adjustment. The obtained parameters (Supplementary Table 4) were tested to replicate the expression of several clock genes under the *lwd1 lwd2* mutant condition (Supplementary Fig. 1a).

Next, we tried to modify the Pokhilko model again so that LWD1 activates both *CCA1* and *PRR9* genes. Therefore, in addition to equation (12) above, we also needed to modify the equation for *CCA1* mRNA. In the Pokhilko model, *CCA1* mRNA was represented as equation (13). Then, equation (13) was modified to

equation (14). Here, we removed parameter n1 and replaced it with three additional parameters to facilitate the LWD1 activation to *CCA1*. The additional parameters were searched as described for equation (12) (Supplementary Table 4). Both equations (12) and (14) were used to describe *CCA1* and *PRR9* mRNA (Supplementary Fig. 1b).

$$\frac{dC_L^m}{dt} = L.q1.C_p + n_1 \frac{g_1^a}{g_1^a + (C_{p9} + C_{p7} + C_{NI} + C_T)^a} - (m_1 L + m_2 D) C_L^m \qquad (13)$$

$$\frac{dC_L^m}{dt} = L.q1.C_p + \left(n_{basal} + n_{lwd} \frac{lwd^2}{lwd^2 + K_{lwd}^2}\right) \frac{g_1^a}{g_1^a + (C_{p9} + C_{p7} + C_{NI} + C_T)^a} - (m_1 L + m_2 D) C_L^m \qquad (14)$$

Supplementary References

1. Mizoguchi T*, et al.* LHY and CCA1 are partially redundant genes required to maintain circadian rhythms in Arabidopsis. *Developmental cell* **2**, 629-641 (2002).

2. Matsushika A, Makino S, Kojima M, Mizuno T. Circadian waves of expression of the APRR1/TOC1 family of pseudo-response regulators in Arabidopsis thaliana: insight into the plant circadian clock. *Plant & cell physiology* **41**, 1002-1012 (2000).

3. Makino S, Matsushika A, Kojima M, Oda Y, Mizuno T. Light response of the circadian waves of the APRR1/TOC1 quintet: when does the quintet start singing rhythmically in Arabidopsis? *Plant & cell physiology* **42**, 334-339 (2001).

4. Pokhilko A, Fernandez AP, Edwards KD, Southern MM, Halliday KJ, Millar AJ. The clock gene circuit in Arabidopsis includes a repressilator with additional feedback loops. *Molecular systems biology* **8**, 574 (2012).

5. Kim JY, Song HR, Taylor BL, Carre IA. Light-regulated translation mediates gated induction of the Arabidopsis clock protein LHY. *The EMBO journal* **22**, 935-944 (2003).

6. Ito S, Nakamichi N, Matsushika A, Fujimori T, Yamashino T, Mizuno T. Molecular dissection of the promoter of the light-induced and circadian-controlled APRR9 gene encoding a clock-associated component of Arabidopsis thaliana. *Bioscience, biotechnology, and biochemistry* **69**, 382-390 (2005).

7. Locke JC*, et al.* Extension of a genetic network model by iterative experimentation and mathematical analysis. *Molecular systems biology* **1**, 2005 0013 (2005).

8. Pokhilko A*, et al.* Data assimilation constrains new connections and components in a complex, eukaryotic circadian clock model. *Molecular systems biology* **6**, 416 (2010).

9. Wang Y, Wu JF, Nakamichi N, Sakakibara H, Nam HG, Wu SH. LIGHT-REGULATED WD1 and PSEUDO-RESPONSE REGULATOR9 form a positive feedback regulatory loop in the Arabidopsis circadian clock. *The Plant cell* **23**, 486-498 (2011).
